# Garfield’s Religious Views

**Published:** 1881-12

**Authors:** E. V. Smalley


### GARFIELD'S RELIGIOUS VIEWS.

E. V. SMALLEY, IN THE CENTURY FOR DECEMBER.

By the natural bent of his mind, the late President had a liking for philosophic and religious studies, which was strengthened and gratified during his two terms at Williams College, where a good deal of attention is given to metaphysics, and his subsequent four years of teaching at Hiram ; but in his late career the practical questions of life absorbed him so much that he found little time to devote to the domain of speculation and theoretical thought. He read Mill, Comte and Spencer, however, and was deeply interested in such books as James Freeman Clarke's "Ten Great Religions," and in the current discussions in the English reviews and magazines on new phases of religious belief and criticisms. There was nothing of the bigot about him. He welcomed all honest discussion, and was always willing to throw off old opinions if convinced they were erroneous. In his religious views he might have been called a rationalistic Christian. I doubt if he could have passed a successful catechising on the doctrinal points of an orthodox creed, but on such essential matters as a belief in the divine guidance of the universe and the immortality of the human soul his faith was unshaken. Modern materialism made no impression upon him. The argument that the mind is only a phenomenon of matter he thought a stupid reversal of the truth. The soul or life-principle was the real thing, he maintained, and the phases of matter only its transient and varying expression. The church to which he belonged from boyhood has no written creed and does not question its members as to their theological conceptions ; therefore he was not hampered by formal statements of faith in his intellectual growth, and was able without hypocrisy to retain associations which became very dear to him in early life.

